# Disseminated *Exophiala dermatitidis* causing septic arthritis and osteomyelitis

**DOI:** 10.1186/s12879-018-3171-0

**Published:** 2018-06-04

**Authors:** Raynell Lang, Jessica Minion, Stuart Skinner, Alexander Wong

**Affiliations:** 1 Department of Medicine, University of Calgary, Calgary, Alberta Canada; 2 Regina Qu’Appelle Health Region, Department of Laboratory Medicine, University of Saskatchewan, College of Medicine, Regina, Saskatchewan Canada; 3 Division of Infectious Diseases, Department of Medicine, University of Saskatchewan, Regina, Saskatchewan Canada; 4 4E - ID Clinic, Regina General Hospital, 1440 14th Avenue Regina, Regina, Saskatchewan Canada

**Keywords:** *Exophiala dermatitidis*, Phaeohyphomycosis, Wangiella dermatitidis, Disseminated, Septic arthritis, Osteomyelitis

## Abstract

**Background:**

*Exophiala dermatitidis* is a melanized fungus isolated from many environmental sources. Infections caused by *Exophiala* species are typically seen in immunocompromised hosts and manifest most commonly as cutaneous or subcutaneous disease. Systemic infections are exceedingly rare and associated with significant morbidity and mortality

**Case presentation:**

A 28-year-old female originally from India presented with fevers, chills, weight loss and increasing back pain. She had a recent diffuse maculopapular rash that resulted in skin biopsy and a tentative diagnosis of sarcoidosis, leading to administration of azathioprine and prednisone. An MRI of her spine revealed a large paraspinal abscess requiring surgical intervention and hardware placement. Cultures from the paraspinal abscess grew a colony of dark pigmented mold. Microscopy of the culture revealed a melanized fungus, identified as *Exophiala dermatitidis.* Voriconazole was initially utilized, but due to relapse of infection involving the right iliac crest and left proximal humerus, she received a prolonged course of amphotericin B and posaconazole in combination and required 7 separate surgical interventions. Prolonged disease stability following discontinuation of therapy was achieved.

**Conclusions:**

Described is the first identified case of disseminated *Exophiala dermatitidis* causing osteomyelitis and septic arthritis in a patient on immunosuppressive therapy. A positive outcome was achieved through aggressive surgical intervention and prolonged treatment with broad-spectrum antifungal agents.

## Background

*Exophiala dermatitidis* is a melanized fungus isolated from many environmental sources [1]. It is a saprobe, using extracellular digestion to obtain nutrients from dead or decaying organic matter [2, 3]. Infections caused by *Exophiala* species are known as phaeohyphomycosis, due to the appearance of dark pigmented, irregular branching, septate hyphae [1–5]. Infections are typically seen in immunocompromised hosts such as transplant recipients and manifest as subcutaneous disease [6, 7]. Systemic infections are rare and associated with significant morbidity and mortality [5, 8, 9].

We describe, to our knowledge, the first case of disseminated *E. dermatitidis* with widespread involvement including osteomyelitis and septic arthritis in a previously healthy 28-year-old female on immunosuppressive therapy. A positive outcome was achieved through aggressive surgical intervention and prolonged treatment with broad-spectrum antifungal agents.

## Case presentation

A previously healthy 28-year-old female presented with complaints of increasing back pain and fevers. Originally from India, she had immigrated to Canada two years prior and within months of arrival, developed fevers associated with unintentional weight loss, and a diffuse rash localized to her torso, legs and scalp. The rash was annular, with dark-colored plaques and central clearing. Skin biopsy identified granulomatous inflammatory cell infiltrates with staining negative for mycobacteria and fungus. A diagnosis of sarcoidosis was suggested. The lesions resolved spontaneously within six months with no specific therapy, but her fevers and weight loss continued.

The patient elected to return to India for further investigations and treatment of her ongoing symptoms. Diffuse lymphadenopathy was identified and an inguinal lymph node biopsy revealed non-caseating granulomatous changes. Mycobacterial and fungal stains were negative. Brucella IgM and IgG serology were positive, but serum agglutination testing (SAT) to detect antibodies against the smooth lipopolysaccharide (S-LPS) of the outer membrane was negative. While in India, she received empiric treatment for tuberculosis and brucellosis with isoniazid, rifampin, pyrazinamide, ethambutol and injectable streptomycin. After nearly eight weeks of therapy, she experienced no clinical change. Her antimicrobials were discontinued, and she was started on high dose prednisone with rapid resolution of her malaise and fevers. She returned to Canada with a presumptive diagnosis of sarcoidosis on hydroxychloroquine, azathioprine, and tapering prednisone initiating at 40 mg daily.

Shortly after her return, the patient developed a large nodule on her back with progressive back pain, worsening left arm pain, and ongoing fevers. She was admitted to hospital for further testing. Hepatosplenomegaly was noted with no appreciable lymphadenopathy. A tender mass was identified medial to her left scapula. She had no focal neurologic deficits and the remainder of her physical exam was non-contributory.

Investigations revealed a white blood cell (WBC) count of 14.3 × 10^9^ per liter (L) and a platelet count of 885 × 10^9^/L. Her liver enzymes and creatinine kinase were within normal limits. Computed tomographic (CT) scanning of the spine showed a large heterogeneously attenuating area in the paraspinal tissue at the level of T3-T4 along with a large nodule in the left upper lung. Bone scan revealed abnormalities in the thoracic spine with increased uptake in the right sacroiliac (SI) joint. The differential diagnosis included sarcoidosis, tuberculosis, brucellosis, and disseminated fungal infection. Further investigations were pursued to identify the causal etiology.

The lung nodule was biopsied and smear-negative for acid-fast bacilli, and culture negative for fungus, bacteria, and mycobacteria. Histopathology reported caseating granulomas and fungal elements resembling budding yeast. A CT scan of her head revealed no abnormalities. Bronchoalveolar lavage (BAL) was performed. *Mycobacterium tuberculosis complex* polymerase chain reaction (PCR) and mycobacterial cultures were negative. Magnetic resonance imaging (MRI) revealed a large soft tissue mass at the spinal levels of T3-T5. Incision and drainage of this large paraspinal abscess was performed, along with a laminectomy at T4 and T5 with rod and pedicle screw placement due to involvement of the adjacent vertebral bodies. Liposomal amphotericin B was started empirically, as the only etiologic clue remained the yeast-like elements identified in the lung nodule biopsy.

**Fig. 1:**
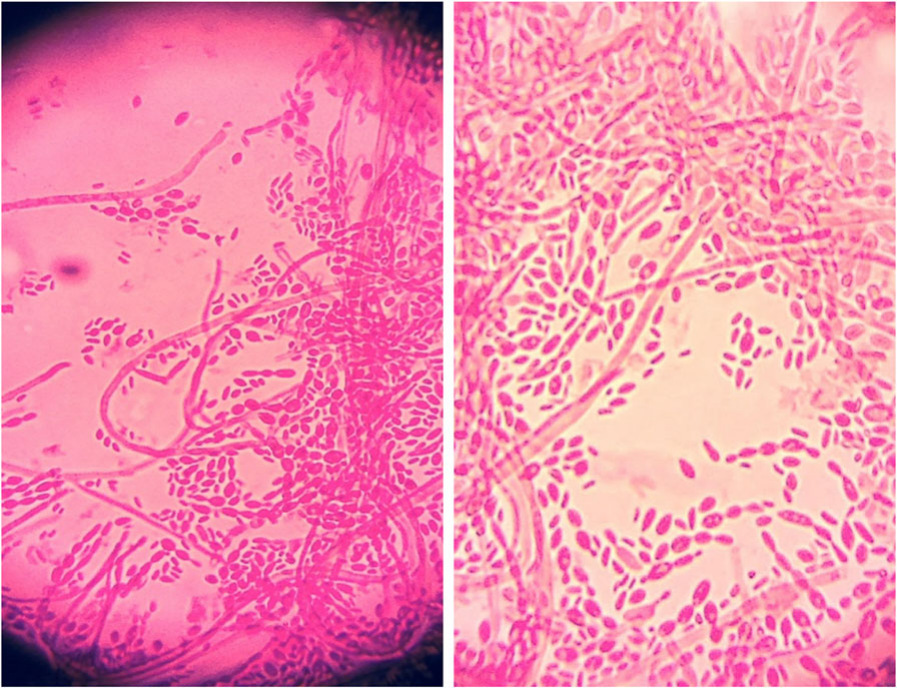
Exophiala dermatitidis isolated from surgically debrided paravertebral tissue. Lactofuchsin mount, 400X

**Fig. 2:**
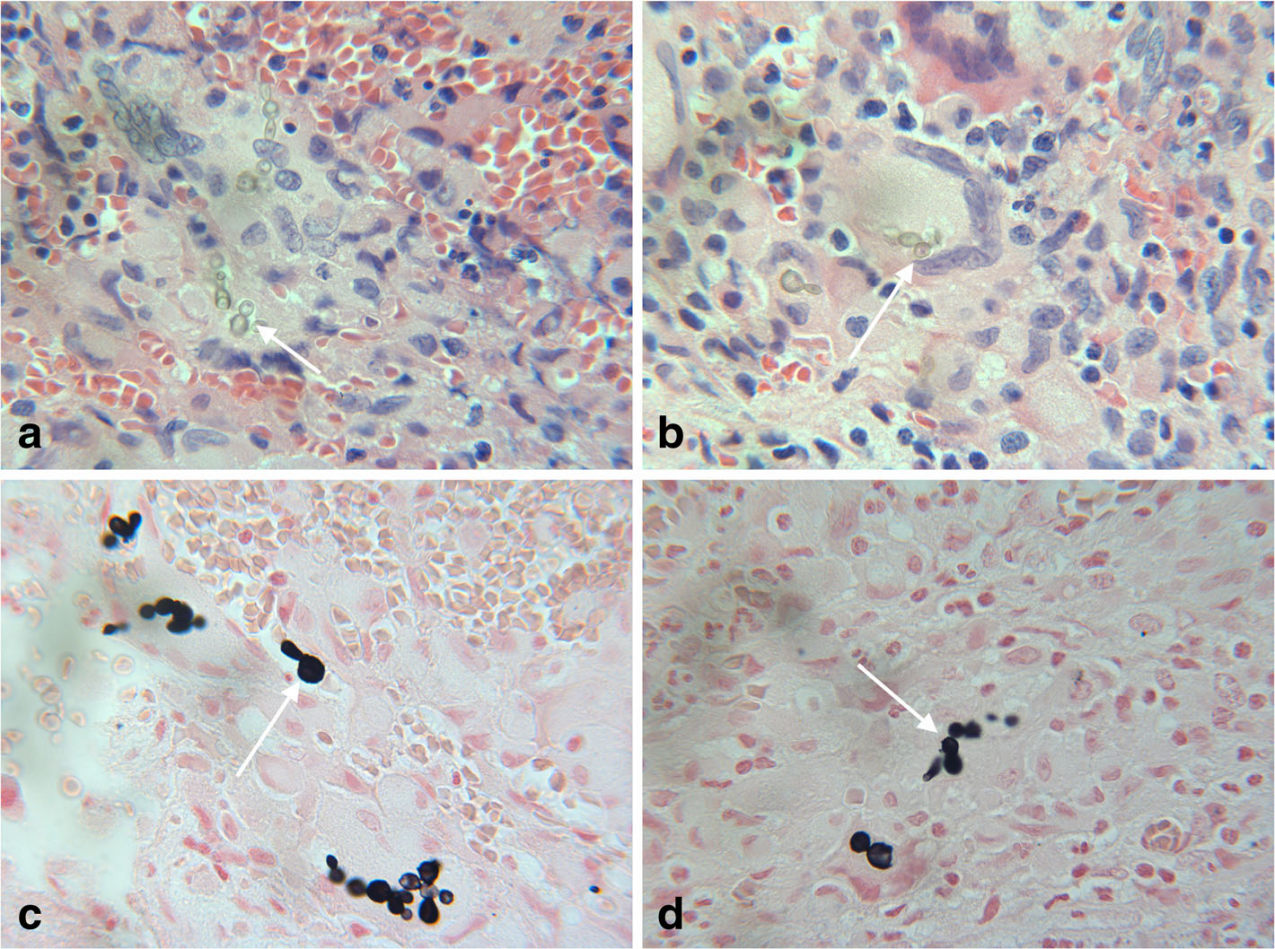
**a**, **b** Fungal elements visualized in surgically debrided paravertebral tissue, identified from culture as Exophiala dermatitidis. H&E staining, 400X. **c**, **d** Fungal elements visualized in surgically debrided paravertebral tissue, identified from culture as Exophiala dermatitidis. Fontana-Masson stain, 400X

Histopathology from the paravertebral collection returned positive for yeast-like cells on PAS and GMS staining, suggestive of histoplasmosis. Bacterial cultures, originally submitted for Brucella, were found to be growing a colony of dark pigmented mold. Microscopy revealed numerous oval shaped conidia with rectangular phialides in keeping with a melanized fungus, identified as *Exophiala* species (Fig. 1). Fontana Masson staining confirmed the presence of pigmented fungi in original pathology specimens (Fig. 2). Voriconazole was added empirically and liposomal amphotericin B was continued. The patient developed progressive pain in her left shoulder and right SI joint. MRI of the pelvis revealed progression of an osteolytic lesion in the right iliac crest and fluid accumulation in the right sacro-iliac joint. Her therapy was changed from voriconazole to posaconazole empirically at 300 mg IV daily given her ongoing disease progression.

Multiple operative cultures and the BAL sample became positive for dark pigmented mold. Sequencing of the D1-D2 region of the large (28S) ribosomal subunit of the isolate resulted in 98.6% similarity to *Exophiala dermatitidis*, confirming a diagnosis of *Exophiala dermatitidis* [10]. Susceptibility testing results are listed in Table 1. Due to local availability of oral antifungals, posaconazole was changed to oral voriconazole 200 mg PO, and liposomal amphotericin B was discontinued following one month of combination therapy given the patient had experienced significant clinical improvement. Prior to discharge from hospital, a trough voriconazole level was found to be therapeutic at 2.99 mcg/mL (target range 2–5 mcg/mL).

**Table 1:** Antifungal Susceptibility Testing of Exophiala dermatitidis isolate

Antifungal	MIC (mg/L)
5-Flucytosine	0.12
Amphotericin B	0.5
Itraconazole	0.25
Posaconazole	0.12
Voriconazole	0.12

Three weeks following discharge, she again presented with an enlarging mass over her left shoulder blade and ultrasound revealed a thick-walled hyper-dense lesion. A spontaneously draining sinus from her thoracolumbar incision was noted. Repeat MRI of her spine and pelvis revealed a bone marrow signal in the right iliac region (7.4 × 2.8 cm) and large peripherally enhancing fluid collections extending bilaterally down the spine, enveloping the spinal hardware. She was readmitted for irrigation and debridement of these collections. Voriconazole was discontinued, and combination therapy with liposomal amphotericin B 200 mg IV daily and posaconazole 300 mg IV daily was resumed.

She continued to have daily fevers with worsening pain and effusion of her left shoulder. MRI demonstrated dramatic changes suggestive of a highly aggressive process involving the right iliac crest and left proximal humerus. Her spinal hardware was removed and the surrounding area extensively debrided along with an excisional arthroplasty of the left shoulder. Operative cultures remained negative despite extended incubation. She remained on posaconazole and liposomal amphotericin B throughout hospitalization and received a six-week course of combination therapy. In total, she required 7 surgical interventions.

Two weeks following discharge, liposomal amphotericin B was discontinued, and posaconazole was continued at 400 mg orally once daily. After receiving nearly 500 days of antifungal therapy following her last surgery, with no clinical evidence of infection and normalized lab parameters, her posaconazole was discontinued. Over 1.5 years following discontinuation of antifungal therapy, she remains disease free with normalized inflammatory markers and continues to be followed closely by the infectious disease team. She has successfully received a shoulder prosthesis to regain significant function in her left arm.

## Discussion and conclusions

Several different species of *Exophiala* have been documented, with *E. dermatitidis* being known to cause cutaneous and subcutaneous phaeohyphomycosis [7]. Systemic infections are rare with an extensive literature review in 2013 documenting 40 cases [5, 8, 9]. Despite identification of *E. dermatitidis* in environmental samples globally, disseminated human infection is extremely rare in North America and nearly all cases are reported from Asia [7, 9]. Environmental samples have been noted in abundance in public steam baths, and water reservoirs, which may relate to the geographic differences in infection [11]. In this case, it is believed that the patient contracted the infection while residing in India. Extra-cutaneous manifestations include lymphadenitis, fungemia, cerebral infections, stomatitis, otitis media, corneal ulcers, esophagitis, pneumonia, liver cirrhosis, pancreatitis, inflammation of the gastrointestinal and biliary systems, endocarditis and peritonitis [2, 4, 9, 12]. Li et al. published a case series of seven fatal *Exophiala sp.* infections in China, two of which had bone involvement, however speciation revealed *Exophiala spinifera* as the etiologic agent [13]. *E. dermatitidis* has not previously been documented to cause bone or joint involvement.

Of all reported cases, approximately 70% have an identifiable underlying risk factor such as an underlying immunocompromised state, intravenous drug use, long-term catheters, malignancy or cystic fibrosis [1, 9]. Case reports reveal that patients often present with hepatomegaly and lymphadenopathy [3, 5, 7]. Biopsies of liver and lymph nodes frequently show granulomas, which may lead to an early misdiagnosis of sarcoidosis, lymphoma or tuberculosis [2, 3].

Most infections initially involve the skin and are suspected to spread hematogenously [7]. The initial skin lesions our patient presented with may have represented cutaneous or subcutaneous fungal infection. Several case reports have described patients being diagnosed with tinea versicolor prior to dissemination with *E. dermatitidis*, leading to the belief that inoculation occurred initially through the skin [3–5]. Since initial biopsies revealed non-specific granulomatous changes, empiric steroids and immunosuppressive agents initiated for sarcoidosis may have facilitated further dissemination of the organism.

*E. dermatitidis* grows slowly and appears only after prolonged incubation of up to seven days, with one study suggesting cultures be held for at least four weeks [2, 14]. The organism may be missed if fungal cultures are not performed. Accurate identification of *Exophiala* species is enabled by molecular diagnostics involving sequencing of the internal transcribed space (ITS) region of the ribosomal DNA [6].

A review of 20 disseminated infections from 1960 to 1992 revealed a mortality rate of 48%, however a subsequent review between 1993 and 2011 based on 24 cases revealed a mortality rate of 25% [1, 5]. Improved outcomes are believed to be due to the availability of improved antifungal therapies. If CNS involvement develops, the survival rate is 20% [1].

No large-scale controlled analyses of treatment options are available. Amphotericin B, flucytosine, itraconazole, voriconazole and posaconazole have all been used with varying degrees of success [2, 15]. In 2011, Badali et al. performed a study comparing 11 different antifungal drug susceptibilities to *E. dermatitidis* and noted that the MICs were similar between environmental and clinical strains of the organism as well as isolates from Asia, America and Europe [16]. The lowest MICs were observed to posaconazole and itraconazole followed by voriconazole [15, 16]. Fluconazole and amphotericin B appear to have poor activity against *E. dermatitidis* [2, 14, 16].

Combination therapy of caspofungin with voriconazole, amphotericin or itraconazole may facilitate synergistic activity against *E. dermatitidis*, although monotherapy with an echinocandin is not recommended [14, 16]. Both voriconazole and posaconazole have good cerebral penetration, ideal for treating disseminated infections. There is limited data regarding the in vivo efficacy of antifungal therapy and there are no defined breakpoints available for antifungal agents [17]. In deep-seated infections, long-term survival has only been reported when complete surgical resection is obtained, however prognosis remains poor [15, 17]. The decision to discontinue therapy was based on demonstrated clinical stability, normalized lab parameters, expert opinion, and extensive discussion with the patient and her immediate family members.

### Key messages

1. Described is the first identified case of disseminated *Exophiala dermatitidis* causing osteomyelitis and septic arthritis in a patient on immunosuppressive therapy.
2. *Exophiala dermatitidis* is a dematiaceous fungus isolated from environmental sources.
3. Systemic infections are very rarely caused by *E. dermatitidis*, however when present are associated with significant morbidity and mortality.
4. *Exophiala dermatitidis* grows slowly and therefore fungal cultures need to be held for prolonged incubation or the organism may be missed.
5. Through aggressive surgical intervention and prolonged antifungal therapy, a positive outcome may be achieved in disseminated *E. dermatitidis* infections.

## Abbreviations

BAL: Bronchoalveolar lavage
CT: Computed tomography
GMS: Grocott’s methenamine silver stain
ITS: Internal transcribed space
MIC: Minimum inhibitory concentration
MRI: Magnetic resonance imaging
PAS: Periodic acid-Schiff stain
PCR: Polymerase chain reaction
SAT: Serum agglutination testing
SI: Sacroiliac joint
S-LPS: Smooth lipopolysaccharide

## References

[1] Suzuki K, Nakamura A, Fujieda A, Nakase K, Katayama N (2012). Pulmonary infection caused by Exophiala dermatitidis in a patient with multiple myeloma: A case report and a review of the literature. Med Mycol Case Rep.

[2] Alabaz D, Kibar F, Arikan S, Sancak B, Celik U, Aksaray N (2009). Systemic phaeohyphomycosis due to Exophiala (Wangiella) in an immunocompetent child. Med Mycol.

[3] Oztas E, Odemis B, Kekilli M, Kurt M, Dinc BM, Parlak E (2009). Systemic phaeohyphomycosis resembling primary sclerosing cholangitis caused by Exophiala dermatitidis. J Med Microbiol.

[4] Hiruma M, Kawada A, Ohata H, Ohnishi Y, Takahashi H, Yamazaki M (1993). Systemic phaeohyphomycosis caused by Exophiala dermatitidis. Mycoses.

[5] Matsumoto T, Matsuda T, McGinnis MR, Ajello L (1993). Clinical and mycological spectra of Wangiella dermatitidis infections. Mycoses.

[6] Silva WC, Gonçalves SS, Santos DW, Padovan AC, Bizerra FC, Melo AS (2017). Species diversity, antifungal susceptibility and phenotypic and genotypic characterisation of Exophiala spp. infecting patients in different medical centres in Brazil. Mycoses.

[7] Kenney RT, Kwon-Chung KJ, Waytes AT, Melnick DA, Pass HI, Merino MJ (1992). Successful treatment of systemic Exophiala dermatitidis infection in a patient with chronic granulomatous disease. Clin Infec Dis.

[8] Chen M, Zhang J, Dong Z, Wang F (2016). Cutaneous phaeohyphomycosis caused by Exophiala dermatitidis: A case report and literature review. Indian J Dermatol Venereol Leprol.

[9] Patel AK, Patel KK, Darji P, Sinqh R, Shivaprakash MR, Chakrabarti A (2013). Exophiala dermatitidis endocarditis on native aortic valve in a postrenal transplant patient and review of literature on E.Dermatitidis Infections. Mycoses.

[10] Clinical and Laboratory Standards Institute (CLSI). Interpretive Criteria for Identification of Bacteria and Fungi by DNA Target Sequencing, 1st Edition. CLSI document MM18-A. 2008. https://clsi.org/standards/products/molecular-methods/documents/mm18/. Accessed 23 Oct 2017.

[11] Matos T, de Hoog GS, de Boer AG, de Crom I, Haase G (2002). High prevalence of the neurotrope Exophiala dermatitidis and related oligotrophic black yeasts in sauna facilities. Mycoses.

[12] Gold WL, Vellend H, Salit IE, Campbell I, Summerbell R, Rinaldi M (1994). Successful treatment of systemic and local infections due to Exophiala species. Clin Infect Dis.

[13] Li DM, Li RY, de Hoog GS, Sudhadham M, Wang DL (2011). Fatal Exophiala infections in China, with a report of seven cases. Mycoses.

[14] Horre R, Schaal KP, Siekmeier R, Sterzik B, de Hoog GS, Schnitzler N (2004). Isolation of fungi, especially Exophiala dermatitidis, in patients suffering from cystic fibrosis. A prospective Study. Respiration.

[15] Sun Y, Liu W, Wan Z, Wang X, Li R (2011). Antifungal activity of antifungal drugs, as well as drug combinations against Exophiala dermatitidis. Mycopathologia.

[16] Badali H, De Hoog GS, Sudhadham M, Meis JF (2011). Microdilution in vitro antifungal susceptibility of Exophiala dermatitidis a systemic opportunist. Med Mycol.

[17] Zheng JS, Sutton DA, Fothergill AW, Rinaldi MG, Harrack MJ, de Hoog GS (2007). Spectrum of clinically relevant Exophiala species in the United States. J Clin Microbiol.

